# Genetic Correlates of Synergy Mechanisms of Daptomycin Plus Fosfomycin in Daptomycin-Susceptible and -Resistant Methicillin-Resistant *Staphylococcus aureus* (MRSA)

**DOI:** 10.3390/microorganisms13071532

**Published:** 2025-06-30

**Authors:** Warren E. Rose, Selvi C. Ersoy, Wessam Abdelhady, Alan R. Dominguez, Jedidiah Ndam Muyah Manna, Jorge N. Artaza, Reetakshi Mishra, Ahmed M. Elsayed, Richard A. Proctor, Sarah L. Baines, Benjamin P. Howden, Nagendra N. Mishra

**Affiliations:** 1 School of Pharmacy, University of Wisconsin-Madison, Madison, WI 53705, USA; 2 Division of Infectious Diseases, The Lundquist Institute at Harbor-UCLA Medical Center, Torrance, CA 90502, USA; 3 David Geffen School of Medicine, University of California, Los Angeles (UCLA), Los Angeles, CA 90095, USA; 4 Department of Health and Life Sciences, Charles R. Drew University of Medicine and Science, Los Angeles, CA 90059, USA; 5 Cypress High School, Cypress, CA 90630, USA; 6 Departments of Medicine and Medical Microbiology & Immunology, University of Wisconsin School of Medicine and Public Health, Madison, WI 53705, USA; 7 Department of Microbiology & Immunology, The University of Melbourne at the Doherty Institute for Infection and Immunity, Melbourne, VIC 3000, Australia; 8 Centre for Pathogen Genomics, The University of Melbourne, Melbourne, VIC 3010, Australia

**Keywords:** MRSA, daptomycin, fosfomycin, combination therapy, transcriptomics

## Abstract

This study elucidates potential genetic determinants and mechanisms involved in the synergistic effects of daptomycin (DAP) + fosfomycin (FOF) combination therapy. Among 33 clinically derived DAP-susceptible (S)/DAP-resistant (R) isogenic strain pairs, mutations in the *mprF* gene occurred in 30/33 DAP-R strains, including polymorphisms of L826F (33%) or T345A/L/I (15%). Strain variants of DAP-S CB1483 serially passaged in vitro for 10 days in DAP +/− FOF identified a key non-synonymous mutation in *mprF* (L826F) only in the DAP monotherapy arm. Interestingly, passage in FOF alone or DAP + FOF prevented the emergence of this *mprF* mutation following 10-day passage. This L826F mprF polymorphism, associated with a “gain-in-function” phenotype, exhibited increased amounts of lysyl-phosphatidylglycerol (L-PG) in the cell membrane (CM). Transcriptomics revealed a relatively modest number (~10) of distinct genes that were significantly up- or downregulated (≥2 log fold) in both the DAP-S and DAP-R strain pairs upon DAP + FOF exposures (vs. DAP or FOF alone). Of note, DAP + FOF decreased expression of *lrgAB* and *sdrE* and increased the expression level of *fosB*. In a rabbit infective endocarditis (IE) model, the DAP-R CB185 strain treated with DAP +/− FOF showed significantly reduced *lrgB* expression in vegetations compared with DAP treatment alone. Overall, these findings indicate that DAP + FOF therapy impacts MRSA through multiple specific mechanisms, enhancing bacterial clearance.

## 1. Introduction

*Staphylococcus aureus* is a prominent human pathogen that causes severe, invasive infections, including infective endocarditis (IE) [1]. Methicillin-resistant *S. aureus* (MRSA) in IE reduces the treatment options wherein vancomycin remains the primary treatment, with daptomycin (DAP) or ceftobiprole as FDA-approved options [2,3,4]. DAP has rapid bactericidal activity against many gram-positive pathogens, including MRSA. While DAP has been used to treat MRSA as an essential standard-of-care antibiotic in bacteremia/IE, DAP-resistance (DAP-R) has emerged during treatment, resulting in treatment failure [4,5]. DAP-R in *S. aureus* is commonly associated with specific genetic mutations in the multipeptide resistance factor (*mprF*) gene, which mediates the gain-in-function lysinylation of phosphatidylglycerol (PG) in the cell membrane (CM), to form lysyl-PG (L-PG) [6,7,8,9,10]. This modifies the cell membrane order and surface charge of *S. aureus* [10,11,12,13,14,15,16]. Factors such as high bacterial inocula, reduced metabolic activity, and altered antibiotic penetration into deep-seated infection sites provide conditions for DAP-R to emerge via this mechanism during treatment [5,17,18].

Innovative approaches are needed to bolster and sustain the efficacy of antibiotics. This could involve using combination therapy strategies with DAP, which have demonstrated success in vitro, in vivo, and in clinical settings [19,20,21,22]. Recently, the combination of DAP + fosfomycin (FOF) has been explored as an experimental approach against *S. aureus* [23,24]. This combination has shown significant bactericidal synergy against MRSA strains in vitro and in experimental IE animal models [23,24]. FOF is a potent bactericidal antibiotic against a broad spectrum of both Gram-positive and Gram-negative pathogens, including multi-drug-resistant strains [25]. Like β-lactams, FOF targets bacterial synthesis of cell wall peptidoglycan [25]. However, it acts by inhibiting an earlier, initial step in peptidoglycan biosynthesis, particularly by blocking UDP-N-acetylglucosamine enolpyruvyl transferase (MurA) [25]. The combination of DAP + FOF was investigated in a small (n = 167 patients, 82 receiving DAP + FOF; 85 DAP alone), randomized, open-label, multicenter clinical trial of MRSA bacteremia in Spain [20]. This combination exhibited a 12% higher rate of treatment success compared with DAP alone, but it did not reach statistical significance for this outcome. Notably, DAP + FOF demonstrated significant secondary endpoints including lower microbiologic failure and lower risk of complicated bacteremia [20].

The molecular mechanisms by which the combination of DAP + FOF exhibits an enhanced impact on MRSA clearance remain largely unknown. Recently, we investigated the phenotypic mechanism by which the addition of FOF to DAP (i) synergistically kills MRSA; (ii) forestalls evolution of resistance to both antibiotics, and/or (iii) resensitizes DAP-R or FOF-R strains to susceptible phenotypes [23]. These effects are intriguing, but the genetic mechanisms of the putative interrelatedness of these three key phenotypic outcome “signatures” against MRSA remain unknown.

This current study uses clinically derived, isogenic DAP-susceptible (DAP-S)/DAP-R MRSA strain pairs to elucidate the potential genetic determinants and mechanisms involved in DAP + FOF combination effects. Integrating genomics and transcriptomics, phenotypic associations, and in vivo expression, we identify key *S. aureus* targets related to DAP +FOF combinatorial effects.

Note: We have used the terminology, “DAP-R”, instead of “DAP-nonsusceptibility” for a more facile presentation.

This work was presented in part at the ESCMID Meeting, Vienna, 11–15 April 2025.

This work is part of a Master’s thesis in Biomedical Sciences by Alan R. Dominguez and Jedidiah Ndam Muyah Manna at Charles R. Drew University of Medicine and Science, Los Angeles, CA, USA.

## 2. Materials and Methods

### 2.1. Bacterial Strains

Clinical MRSA bloodstream isolates of 33 DAP-S and 33 DAP-R isogenic strain pairs were used in this study and have been published elsewhere (Table 1 and Table S1) [26,27,28]. In brief, each pair was collected from a patient with MRSA bacteremia and consists of an initial isolate before DAP treatment (DAP-S) and a subsequent isolate from the same patient that evolved DAP-R [26,27,28]. We employed a well-characterized MRSA pair, CB1483 (DAP-S) and CB185 (DAP-R), for the transcriptomics analysis (Table 2 and Table S2A–F) [27]. The isogenicity of the strain pairs was confirmed by genotypic profiling (i.e., clonal complex, *agr*, *spa*, and *SCC mec* typing) published elsewhere [26,27].

### 2.2. Minimum Inhibitory Concentrations (MICs)

The DAP MIC breakpoint distinguishing DAP-S and DAP-R MRSA isolates is ≤1 mg/L for susceptible and >1 mg/L for resistance [29]. The DAP MIC was performed by Etest, as described previously [29], on Mueller-Hinton agar. FOF susceptibility was measured via broth microdilution MIC testing, with concentrations ranging from 0.25 to 256 mg/L, using cation-adjusted Mueller–Hinton broth (CAMHB) supplemented with 25 mg/mL glucose-6-phosphate (G6P, Sigma-Aldrich Chemical Co., St. Louis, MO, USA). The 5 × 10^5^ CFU/well of the initial inoculum (standardized from a McFarland unit of 0.5) was used, and all incubations were conducted at 37 °C for 16–20 h. The MIC was defined as the lowest antibiotic concentration that inhibited visible growth.

### 2.3. Serial In Vitro Passage Experiments

The DAP-S MRSA strain CB1483 was serially passaged for 10 days in distinct concentrations of DAP, FOF alone, and DAP + FOF to investigate the prevention of emergence of DAP-R or increase in FOF MIC. Passage was performed in CAMHB further supplemented with 50 mg/L calcium (for DAP) and G6P (for FOF) [23]. WGS, described in detail below, pre- and post-passage was performed. Initial concentrations of DAP were 0.5 × MIC (0.125 mg/L for CB1483) and were increased two-fold daily [23,24]. If growth was not observed at a particular concentration, the previous concentration that allowed growth was maintained, and the passaging process was repeated. To prevent excessive escalation of DAP MICs beyond the clinically relevant range for DAP-R *S. aureus* (2–4 mg/L), DAP concentrations did not exceed 4 mg/L [23,24]. For FOF, an initial concentration of 4 mg/L (0.5 × MIC for both strains) was used throughout the passage experiments and remained constant [23,24].

All passage mixtures were incubated at 37 °C for 16–20 h, with a flask-to-medium volume ratio of 7:1 and shaking at 225 rpm [23,24]. Daily passaging involved inoculating 20 µL into 2 mL of fresh medium. After each day’s passage, samples were collected and stored at (−80 °C for subsequent in vitro assays. The proportion of MRSA strains developing a DAP-R phenotype was determined by parallel plating onto Mueller–Hinton agar plates (plus 50 mg/L calcium), with and without DAP at concentrations of 2 and 4 mg/L. Colonies growing on antibiotic screening plates were further evaluated for DAP MICs using Etest [29]. To assess the stability of DAP-R strains that emerged during the 10-day passage period, resulting resistant strains were passaged for an additional 5 days in antibiotic-free CAMHB [23].

### 2.4. CM Phospholipid (PL) Composition

*S. aureus* membrane phospholipid (PL) composition was analyzed as described before [8,9,12,13,14,15,26,28,30]. Major PLs—lysylphosphatidylglycerol (LPG), phosphatidylglycerol (PG), and cardiolipin (CL)—were separated using two-dimensional thin-layer chromatography (2D-TLC) with a specified solvent system [8,9,12,13,14,15].

PL spots on TLC plates were scraped, digested with 70% perchloric acid, and spectrophotometrically quantified at an OD660 using a phosphate estimation assay. PL spot identification was confirmed by iodine vapor exposure and spraying with CuSO_4_ (100 mg/mL) in 8% phosphoric acid, heated at 180 °C [8,9,12,13,14,15].

A ninhydrin staining assay was performed to identify CM-LPG. Each TLC spot was identified by comparison with known positive controls of standard PLs. Data are presented as mean (±SD) percentages of the three major PLs (total LPG + PG + CL = 100%).

### 2.5. Genetic Profiling

To establish genetic mechanisms involved in DAP + FOF interaction dynamics in MRSA, and their relationships to signature phenotypic outcome metrics [23], we performed whole genome sequencing (WGS) and RNA sequencing (comparing pre- and post-DAP + FOF exposures vs. DAP alone or FOF alone exposures).

For whole genome sequencing (WGS), genomic DNA of the studied MRSA strain pairs was extracted prior to and then following DAP +/− FOF exposures from each experiment using the Qiagen QIAampDNA mini-Kit. WGS was performed using the Illumina Nova Seq 6000 for sequencing and reads assembled at the UW-Madison Biotechnology Center DNA Sequencing Facility. The short-read sequence data were mapped to lineage or strain-matched reference genomes for each pair using Snippy v4.5.

For RNA sequencing (RNA-seq)-based transcriptomics, genomic RNA was extracted prior to and following DAP +/− FOF exposures to CB1483 and CB185 MRSA. Strains were growth in antibiotic-free media, DAP 0.5 × MIC, FOF 0.5 × MIC, or DAP + FOF 0.5 × MIC of both antibiotics for 16–20 h. Next, 20 µL of the overnight culture was inoculated into 2 mL of medium conditions above and grown to exponential growth phase (OD_600_ = 0.5), when RNA was extracted using the Qiagen QIAampDNA mini-Kit. Alignment of adapter-trimmed [31,32] 2 × 150 (paired-end; PE) bp strand-specific Illumina reads to the *S. aureus* subsp. *aureus* COL ASM1204v1genome (assembly accession GCA_000012045.1) was achieved with bowtie2 software v2.4.4 [33]. Expression estimation was performed with RSEM v1.3.3 [34]. To compare expression differences among the DAP or FOF treated samples and DAP + FOF treated sample versus untreated cells, expected counts derived from RSEM were log transformed after first filtering the expression matrix from low-count genes (transcript count per million > 1 across both samples) and performing library normalization with the trimmed mean of M-values [34]. The top 100 most variable genes were selected; the expression in each condition was subtracted from the mean for each gene and a heatmap constructed with pHeatmap v1.0.12 [32]. In bioinformatics analysis, we focused on genes “significantly” up- or downregulated (>2 log_2_-fold, comparing control vs. antibiotic-exposed conditions) following DAP + FOF exposures (vs. DAP alone or FOF alone exposures). We used relevant programs (e.g., KEGG; Gene Ontology) to organize differentially expressed genes into the most closely aligned metabolic and other functional groupings.

### 2.6. In Vitro qRT-PCR Validation of the Selected Genes

Following RNA-seq analyses, we performed in vitro qRT-PCR validation of the *lrgB* gene selected from the overall repertoire of the differentially expressed genes. *lrgB* is an anti-holin locus that represses murine hydrolase secretion and yields enhanced autolysis and increased organism clearance. The qRT-PCR was performed using primers listed in Table S3. The *gyrB* gene was used as a housekeeping gene for transcript normalization. Because of the greater antibiotic pressure of the DAP + FOF combination on bacterial growth, bacteria were grown overnight to collect the optimum amount of bacterial cells for the RNA extraction. In brief, for in vitro RNA extraction, cells were grown overnight in brain heart infusion (BHI) in the presence of 1 × MIC of the indicated antibiotic. When cells were exposed to DAP, media was supplemented with 50 µg/mL Ca^2+^. Following overnight growth, cells were pelleted and RNA extracted as previously described [35,36,37]. The qRT-PCR was performed on a StepOne thermocycler (ThermoFisher, Waltham, MA, USA) and analyzed with StepOne Software. Relative gene expression was calculated using the 2^−ΔΔCT^ method, performed in experimental triplicate for each treatment group in at least two independent runs.

### 2.7. Rabbit IE Model and RNA Extraction from In Vivo Rabbit Vegetations

In vivo experiments were performed to confirm the translatability of the observed activity from the in vitro gene expression studies described above. For this purpose, the well-characterized rabbit aortic valve IE model was employed validating the potential capacity of DAP + FOF combination to synergistically kill (>2 log reduction in CFU/g tissue compared to either agent alone) in various target tissues i.e., cardiac vegetations, kidney, and spleen as described before [23]. The IE studies employed the DAP and FOF dose regimens (10 mg/kg and 300 mg/kg i.v., respectively; given once daily for 4 days) in rabbits. The experimental IE employed the DAP-R CB185 MRSA strain receiving (i) no therapy; (ii) DAP alone; (iii) FOF alone; or (iv) DAP + FOF combination at the same dose-regimens as described above. Animals were sacrificed at least 12 h after the last antibiotic doses to minimize antibiotic carryover (based on the half-lives of the DAP or FOF in rabbits Cardiac vegetations, kidneys, and spleens were sterilely excised, weighed, and quantitatively cultured [23]. Vegetation samples from untreated rabbits, or those treated with either DAP, FOF, or combination (DAP + FOF) were pooled by treatment group and homogenized using a gentleMACS Dissociator (Miltenyi Biotech San Jose, CA USA) in RLT buffer (Qiagen) containing 1% β-mercaptoethanol (*v*/*v*) [36] Homogenized vegetations were centrifuged at 2000 rpm for 1 min and the supernatant was collected directly for RNA extraction as described previously [35,36,37,38]. qRT-PCR and analyses were performed as described in in vitro qRT-PCR validation.

### 2.8. Statistical Analysis

Statistical comparisons were performed using the unpaired Student *t*-test and Kruskal–Wallis ANOVA. *p* values < 0.05 were considered statistically significant

## 3. Result and Discussion

### 3.1. Whole Genome Sequencing (WGS)

We performed WGS for 33 DAP-S and 33 DAP-R MRSA strain pairs to establish the genetic determinants in the DAP-R development of each pair. Thirty DAP-R strains contained mutations in the *mprF* locus (involved in maintenance of a relative positive surface charge in *S. aureus* via lysinylation of CM phosphatidylglycerol [PG] to generate lysyl-PG [LPG]) [9,10,13,16]. The most common *mprF* single nucleotide polymorphisms (SNPs) identified resulted in either L826F or T345A/L/I substitutions, constituting 33% and 15% of strains, respectively (Table 1 and Table S1). These SNPs are well-known *mprF* ‘hot spot’ mutations in the open reading frame linked to DAP-R (7–15). In addition, some of the DAP-R strains had mutations among other genes involved in lipid metabolism known for mediating this phenotype in *S. aureus* such as *cls1* and *cls2* (cardiolipin biosynthesis), *yycG*, *fabFH* (maintenance of membrane homeostasis and fatty acid synthesis, respectively), and *crtN* (carotenoid biosynthesis, impacting membrane fluidity/rigidity) (Table 1 and Table S1) [8,11,12]. In addition, in a selected number of strains, mutations were also observed in *dlt* (surface charge maintenance via cell wall d-alanylation); and *vraS* (involved in cationic antimicrobial peptide sense-response) (Table 1 and Table S1) [35].

We next performed serial passage of a strain pair in DAP alone, FOF alone, and DAP + FOF over a 10-day period. This strain pair was selected for passage because the pre- and post-passage DAP MICs were 0.25 mg/L and 4 mg/L, respectively, consistent with typical DAP MICs of these phenotypes in the clinical setting. Also, the DAP-R strain of this pair (CB185) contained a common “hot-spot” *mprF* SNP in L825F, as well as a *cls2* mutation, which are both noted to also be independently associated with DAP-R (Table 2). Furthermore, this strain pair was ideal because both strains exhibited the same susceptibility to FOF (8 mg/L), thereby providing an equal assessment of FOF. WGS identified a limited number of mutations in comparing the three passage groups, mostly in genes of unknown function. However, of importance, a key non-synonymous *mprF* mutation occurred in the DAP alone post-passage group in the synthase domain of the MprF protein (L826F) (Table 2). This replicated the same mutation that occurred in the DAP-R strain (CB185) derived in the clinical setting from DAP treatment. DAP susceptibility testing of this passage strain also found that a similar DAP-R phenotype emerged (MIC = 4 mg/L) by day 10 of passage. Notably, such mutations occur only in a limited number of key “hotspots” within *mprF* (such as the one identified above) and are linked to a “gain-in-function” phenotype, yielding excess amounts of CM L-PG [12,13,15,16]. Passage of the wild-type DAP-S parental strain (CB1483) in FOF alone or DAP + FOF prevented the emergence of the DAP-R phenotype (day 10 passage DAP MIC = 1 mg/L). Further, the DAP + FOF passaged strain displayed a wild-type phospholipid phenotype and did not accumulate SNPs associated with DAP-R following 10 days of passage treatment (Table 2). We previously noted the profound synergistic killing of DAP + FOF in this strain background, and here we identify the impact of FOF on preventing DAP-R. This is notable given the improved microbiologic response of this combination observed in the clinic [20].

The novel findings of above indicate that FOF can forestall the emergence of DAP-R through prevention of mutations associated with increasing outer membrane surface charge (*mprF/cls*). To underscore the key and correlative phenotypic impact of these findings, we quantified the proportionality of the three major CM PLs in the post-passage variants. These PL data of DAP passage alone were compared with the FOF alone and DAP + FOF combination passages. As seen in Table 2, the emergence of the *mprF* mutation in the DAP alone passage variant was clearly a gain-in-function SNP, with increased amounts of CM L-PG detected, coincident with reduced PG levels. In contrast, the two other passage groups (FOF alone and DAP + FOF) that prevented the emergence of this *mprF* mutation contained CM L-PG at parental control levels (Table 2). It is noteworthy that the ratio of the PG/L-PG correlates with *S. aureus* susceptibility not only to DAP but also to key innate host-defense peptides. Therefore, FOF may play a critical role with DAP in strengthening the innate immune response to improve bacterial clearance.

### 3.2. RNA Sequencing (RNA-Seq)-Based Transcriptomics

**Figure 1:**
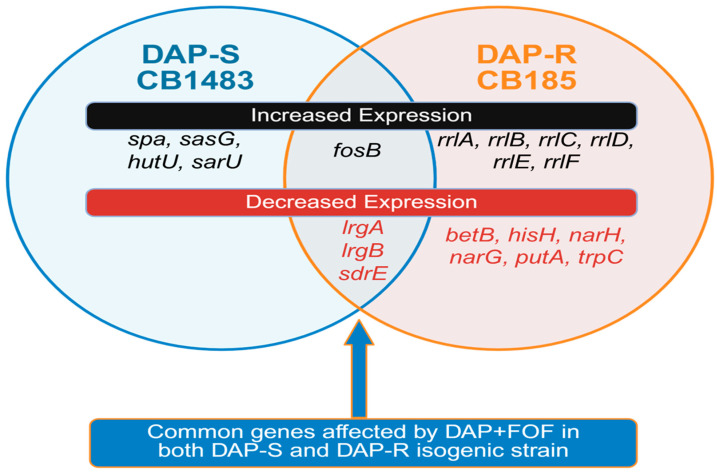
Venn diagram of genes altered ≥ 2-fold by DAP + FOF vs. DAP or FOF alone exposures in DAP-S/DAP-R MRSA strain pair. Red and black fonts indicate whether expression was increased (black) or decreased (red) by DAP + FOF exposure.

We used transcriptomic analysis to identify genes at the transcriptome level that may be mechanistically involved in the response to DAP + FOF treatment. We compared transcriptomic profiles by RNA-seq of DAP-S strain CB1483 and DAP-R strain CB185 without antibiotic, compared with DAP, FOF, or the combination of DAP + FOF at exponential growth as described (Figure 1; Table S2A–F). Transcriptomic analysis revealed differentially and selectively expressed genes in both DAP + FOF-exposed strains; there were a limited number of genes that were differentially (significantly) expressed in both strains. These included upregulation in *fosB* along with downregulation of *lrgA*, *lrgB*, and *sdrE*. The upregulation of *fosB* represents an expected transcriptional response in *S. aureus* containing this gene, as this locus is involved in the reduced FOF susceptibility phenotype [39]. Of interest, *lrgA* was downregulated; *lrgAB* is an anti-holin locus, which represses murine hydrolase secretion, suggesting that DAP + FOF-induced suppression of this locus may yield enhanced autolysis and increased organism clearance [40,41]. This locus is also involved in biofilm formation [41,42], suggesting DAP + FOF-mediated repression of *lrgAB* fosters less biofilm formation, and potentially better clearance of DAP-R strains by antibiotics and host defenses. This is further supported by downregulation of *sdrE* (responsible for adhesion, pathogenicity, and immune evasion) [43]. We are currently investigating the relationship of DAP + FOF-induced downregulation of *lrgAB* and *sdrE*, as adhesion and biofilm formation is controlled by several genetic responses in *S. aureus*.

Several genes-of-interest were differentially regulated in either the DAP-S strain or the DAP-R strain, but not both, following DAP + FOF exposure. In the DAP-S strain, these include upregulation of genes that potentially mediate bacterial clearance (i.e., *sasG* gene, encoding a cell wall surface anchor protein); virulence (i.e., *spa*, encoding protein A, enabling evasion of host immune responses); metabolic pathway genes (i.e., *hutU*, involved in histidine metabolism), and regulatory genes (i.e., *sarU*, a staphylococcal accessory regulator family gene; an activator of *agr* expression). In the DAP-R strain, there were distinct differentially expressed genes observed, including upregulation of the ribosomal locus *rrlA-F* and downregulation of several metabolic pathway genes, amino acid metabolic genes, and stress response genes. This is consistent with the ability of FOF to interfere with the metabolic response in DAP-R strains noted previously (Figure 1) [24].

### 3.3. qRT-PCR Validation of the lrgB Gene

**Figure 2:**
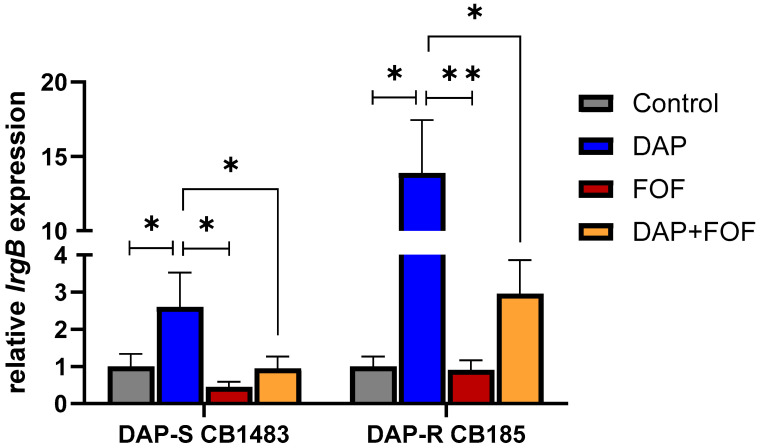
*In vitro lrgB* gene expression was significantly decreased in the DAP-S CB1483 and DAP-R CB185 MRSA strain pairs treated either with FOF alone or DAP + FOF vs. DAP alone treatment. * *p* < 0.05; ** *p* < 0.01 vs. DAP exposure.

We were particularly interested in the downregulation of *lrgAB*, identified in transcriptomic comparison between DAP-S and DAP-R strains set during DAP + FOF treatment. Importantly, this locus is critical in MRSA virulence and pathogenesis, as well as in resistance of this organism to multiple antimicrobial and host defense clearance mechanisms [40,41,42,43]. Therefore, we performed further validation of this gene by qRT-PCR in the same strain pair (CB1483/CB185) following DAP, FOF, and DAP + FOF exposures. For these experiments, we focused on the latter co-impacted gene, *lrgB*, which showed decreased expression following FOF exposures in both strains. In validation studies done in vitro by qRT-PCR, both FOF alone and the DAP + FOF combination blocked DAP-induced hyper-expression of *lrgB* in both strains (Figure 2), consistent with our RNA-seq analyses.

### 3.4. Impacts of DAP-FOF Therapy upon Expression of lrgB Genes in Rabbit IE Vegetations

**Figure 3:**
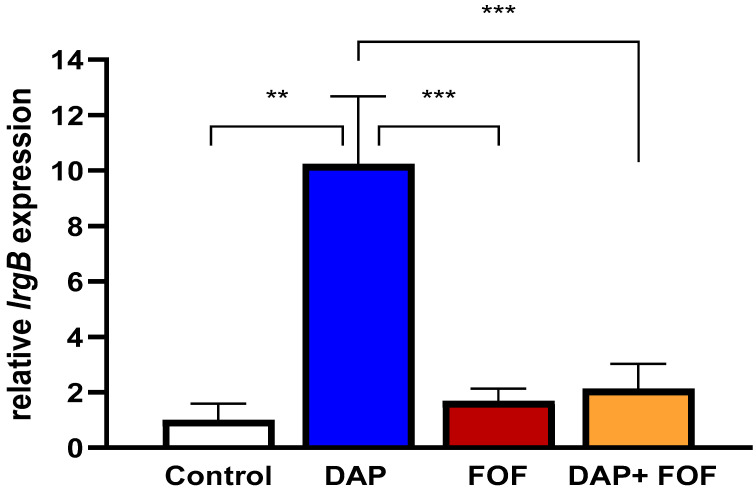
*In vivo lrgB* gene expression was decreased in rabbit endocarditis vegetations of DAP-R CB185 treated either with FOF alone or DAP + FOF or control (untreated) vs. DAP alone. ** *p* < 0.01; *** *p* < 0.001 vs. DAP exposure.

As noted above, FOF exposure in vitro caused repression of the anti-holin gene *lrgB* in both our prototype strain pairs. Therefore, in-parallel, in vivo gene expression experiments were performed to confirm the translatability of the *lrgB* gene expression from the in vitro studies described above. We quantified the intra-vegetation (cardiac vegetation samples isolated from rabbit IE model) expression of *lrgB* by qRT-PCR in rabbits with experimental IE caused by the DAP-R CB185 MRSA strain receiving (i) no therapy; (ii) DAP alone; (iii) FOF alone; or (iv) DAP + FOF combination at the same dose-regimens as reported before [23]. Similar to our in vitro RNA-seq data, FOF alone and DAP + FOF caused substantial reductions in expression of *lrgB* compared to DAP alone, similar to expression levels observed in untreated vegetations (Figure 3). These data suggested that the notable in vivo microbiologic efficacy of DAP + FOF in this model may relate, in part, to impacts on this key regulatory gene. 

## 4. Conclusions

Our genetic analyses provided the validation for the central hypothesis of this investigation that DAP–FOF combinations can impact MRSA through multiple mechanisms. The studies likely reveal consensus genes/pathways most consistently related to the abilities of DAP + FOF combinations to evoke bactericidal synergistic outcomes. The genomic and transcriptomic signatures in the context of DAP + FOF synergy and/or resistance prevention mechanisms indicate that *lrgAB* is a key locus of interest with this combination because it represses murine hydrolase secretion and may increase autolysis. Additional DAP-S/DAP-R strain pairs along with genetic knockout and complementation studies are key to further validating this unique mechanism. These studies are imperative to translate these findings to clinical scenarios and therapeutics regimens for future clinical applications.

## Figures and Tables

**Table 1:** Whole genome sequencing of 33 DAP-S/DAP-R strains. Single nucleotide polymorphisms (SNPs) represent differences compared to the respective isogeneic parent strain (N = 33).

*Polymorphisms in mprF*, Amino Acid Changes (# of DAP-R Strains)	Polymorphisms Among Other Genes (# of DAP-R Strains)
Leu826Phe (11); Thr345Ala/Lys/Ile (5); Ser337Leu (2); Ser295Leu (2); Leu341Ser (4); Met347Arg (2); Val351Glu (1); Thr472Lys (1); Ile420Asn (1); Phe349_Asn352del (1), Pro314Leu (1); Leu42del (1); No mutation (1)	*cls2* (5); *cls1* (1); *dltD* (1); *yycG* (3); *crtN* (1); *lytN* (2); *vraRS* (4); *fabFH* (2)

Parenthesis ( ) = number of DAP-R strains.

**Table 2:** WGS and phospholipid composition of DAP-S1483 strain passaged in DAP or FOF alone and DAP + FOF for 10 days in vitro vs. control (untreated) strain.

Strain	Antibiotic Exposure for 10 Days	DAP MIC (µg/mL)	FOF MIC (µg/mL)	*mprF* or *cls* SNP	% of PL Composition (Mean ± SD)		
					L-PG	PG	CL
DAP-S 1483	Control **	0.25 (S)	8 (S)	None	14 ± 3	76 ± 3	9 ± 1
	DAP	4 (R)	8 (S)	*mprF* (L826F); cls2: (Leu52Phe)	25 ± 6 *	58 ± 9 *	17 ± 6
	FOF	0.5 (S)	>256 (R)	None	12 ± 3	76 ± 9	13 ± 6
	DAP + FOF	1 (S)	8 (S)	None	12 ± 1	74 ± 11	14 ± 12

* *p* < 0.01 vs. control. ** *p* < 0.003; DAP-S vs. DAP-R strain. S = susceptible; R = resistant; CL = cardiolipin.

## Data Availability

The original contributions presented in this study are included in the article/Supplementary Materials. Further inquiries can be directed to the corresponding author.

## Supplementary Materials

The following supporting information can be downloaded at https://www.mdpi.com/article/10.3390/microorganisms13071532/s1. Table S1. Whole genome sequencing of DAP-S/DAP-R MRSA strain pairs; Table S2A. Summary of gene functional classes altered ≥ two-fold in DAP-S 1483 exposed to FOFA; Table S2B. Summary of DAP-S 1483 gene functional classes altered ≥ 2-fold in DAP-S 1483 exposed by DAPA alone; Table S2C. Summary of gene functional classes altered ≥ two-fold in DAP-S 1483 exposed by DAP–FOF vs. DAPA alone; Table S2D. Summary of gene functional classes altered ≥ two-fold in DAP-R 185 exposed by FOFA; Table S2E. Summary of gene functional classes altered ≥ two-fold in DAP-R 185 exposed by DAPA; Table S2F. Summary of gene functional classes altered ≥ two-fold in DAP-R 185 exposed by DAP + FOF vs. DAPA alone; Table S3. Primers used in this study for qRT-PCR gene expression analyses.
