# Supplementary material for: Genetic Correlates of Synergy Mechanisms of Daptomycin Plus Fosfomycin in Daptomycin-Susceptible and -Resistant Methicillin-Resistant Staphylococcus aureus (MRSA)
Source: Microorganisms. 2025 Jun 30;13(7):1532. doi: 10.3390/microorganisms13071532 (PMC12299106; doi:10.3390/microorganisms13071532)
Supplement: Supplementary file 1 [file microorganisms-13-01532-s001.zip › microorganisms-3701488-supplementary.pdf]

Table S1: Whole genome sequencing of DAP-S/DAP-R MRSA strain pairs.

DAP-S (DAP MIC[A])	DAP-R (DAP MIC)	Polymorphisms in DAP-R strain compared to DAP-S parent
C1 (0.19)	C2 (2)	*mprF* (*fmtC*)D: p.Leu826Phe
C3 (0.5)	C4 (4)	*mprF* (*fmtC*): p.Pro314Leu
C5 (0.25)	C6 (3)	*mprF* (*fmtC*): p.Thr345Ala
C7 (0.5)	C8 (3)	*mprF* (*fmtC*): p.Phe349_Asn352del
C9 (0.5)	C10 (3)	*mprF* (*fmtC*): p.Leu826Phe
C13 (0.75)	C14 (4)	*mprF* (*fmtC*): p.Thr472Lys; *cls2*: c.*927T>C; *lytN*: c.*2054C>T; *vraR*: c.-1052A>G; *vraS*: c.-19A>G; *fabF*: c.-4685T>C; *fabH*: c.-3732T>C
C15 (0.75)	C16 (4)	*mprF* (*fmtC*): p.Met347Arg
C17 (0.5)	C18 (4)	*mprF* (*fmtC*): p.Leu341Ser; *cls2*: p.Arg295Cys
C19 (0.38)	C20 (4)	*mprF* (*fmtC*): p.Leu826Phe; *cls2*: p.Ala214Thr; *dltD*: p.Asp350Glu
C22 (0.5)	C23 (4)	*cls1*: p.Lys147Gln;
C24 (0.5)	C25 (3)	*mprF* (*fmtC*): p.Ser295Leu
C26 (0.38)	C27 (2)	*mprF* (*fmtC*): p.Thr345Lys
C30 (0.25)	C31 (2)	*mprF* (*fmtC*): p.Leu826Phe
C32 (0.5)	C33 (2)	*mprF* (*fmtC*): p.Ser337Leu
C36 (0.5)	C37 (3)	*mprF* (*fmtC*): p.Val351Glu; *vraS*: p.Ile240Thr
C38 (0.72)	C39 (3)	*mprF* (*fmtC*): p.Leu826Phe
C40 (0.25)	C41 (3)	*mprF* (*fmtC*): p.Met347Arg
C42 (0.75)	C43 (3)	*mprF* (*fmtC*): p.Ser337Leu; *vraS*; c.-1067C>T
C46 (0.38)	C47 (3)	*mprF* (*fmtC*): p.Leu826Phe
C48 (0.5)	C49 (2)	*mprF* (*fmtC*): p.Thr345Lys
C50 (0.5)	C51 (2)	*mprF* (*fmtC*): p.Thr345Ile
CB1483 (0.25)	CB185 (4)	*mprF* (*fmtC*): p.Leu826Phe; cls2: p.Leu52Phe
CB5079 (0.5)	CB5080 (2)	*mprF* (*fmtC*): p.Leu826Phe
CB5083 (0.25)	CB5082 (4)	*mprF* (*fmtC*): p.Leu341Ser;
CB5088 (0.5)	CB5089 (2-4)	*mprF* (*fmtC*): p.Ser295Leu
CB1631 (0.5)	CB1634 (4)	*mprF* (*fmtC*): p.Leu826Phe; *yycG* (*walK)*: Lys158 frame shift
CB1663 (0.5)	CB1664 (4)	*mprF (fmtC):* p.Leu826Phe*; yycG (walK):* p.Arg86His
CB5057 (0.5)	CB5059 (4)	*mprF* (*fmtC*): p.Ile420Asn
CB5062 (0.5)	CB5063 (8)	*mprF* (*fmtC*): p.Leu341Ser
CB5015 (1)	CB5016 (4)	*mprF_(fmtC):* p. Leu42del; *yycG (walK):* Asn584Ser
J01 (0.5)	J03 (2-4)	*mprF* (*fmtC*): p.Thr345Ile
D592 (0.5)	D712 (2)	*mprF* (*fmtC*): p.Leu341Ser
O325 (0.25)	O510 (2)	*mprF* (*fmtC*): p.Leu826Phe; *cls2*: p.Ala23Val

[A]DAP MICs determined by E-test and indicated as μg/mL

Table S2A. Summary of gene functional classes altered ≥ 2-fold in DAP-S 1483 exposed to FOF[A]

Locus Tag[B]	Gene Name	Expression Level	Pathway Descriptions	Related Pathways
SACOL0137	*cap5B*	Increased	capsular polysaccharide biosynthesis protein (Cell wall & capsule)	Cell wall and capsule synthesis
SACOL0138	*cap5C*	Increased	capsular polysaccharide biosynthesis protein (Cell wall & capsule)	
SACOL0139	*cap5D*	Increased	capsular polysaccharide biosynthesis protein (Cell wall & capsule)	
SACOL0140	*cap5E*	Increased	capsular polysaccharide biosynthesis protein (Cell wall & capsule)	
SACOL0141	*cap5F*	Increased	capsular polysaccharide biosynthesis protein (Cell wall & capsule)	
SACOL0142	*cap5G*	Increased	capsular polysaccharide biosynthesis protein (Cell wall & capsule)	
SACOL0143	*cap5H*	Increased	capsular polysaccharide biosynthesis protein (Cell wall & capsule)	
SACOL0144	*cap5I*	Increased	capsular polysaccharide biosynthesis protein (Cell wall & capsule)	
SACOL0145	*cap5J*	Increased	capsular polysaccharide biosynthesis protein (Cell wall & capsule)	
SACOL0146	*cap5K*	Increased	capsular polysaccharide biosynthesis protein (Cell wall & capsule)	
SACOL0147	*cap5L*	Increased	capsular polysaccharide biosynthesis protein	
SACOL0148	*cap5M*	Increased	capsular polysaccharide biosynthesis galactosyltransferase (Cell wall & capsule)	
SACOL0149	*cap5N*	Increased	capsular polysaccharide biosynthesis protein	
SACOL0150	*cap5O*	Increased	capsular polysaccharide biosynthesis protein (Cell wall & membrane biogenesis)	
SACOL0151	*cap5P*	Increased	UDP-N-acetylglucosamine 2-epimerase – (Cell wall & membrane biogenesis)	
SACOL2632	*cudT*	Decreased	BCCT family osmoprotectant transporter-	Transporter
SACOL2068	*kdpA*	Increased	potassium-transporting ATPase subunit A-	
SACOL2067	*kdpB*	Increased	potassium-transporting ATPase subunit B -	
SACOL2066	*kdpC*	Increased	potassium-transporting ATPase subunit C -	
SACOL2070	*kdpD*	Increased	sensor histidine kinase KdpD -	
SACOL0247	*lrgA*	Decreased	murein hydrolase regulator LrgA -	
SACOL0248	*lrgB*	Decreased	antiholin-like protein LrgB-	
SACOL2176	*opuD2*	Increased	Glycine betaine transporter	
SACOL2340	*gltS*	Decreased	sodium:glutamate symporter	
SACOL0620	*proP*	Decreased	osmoprotectant proline transporter	
SACOL2326	*fosB*	Increased	fosfomycin resistance protein	Cell Envelope
SACOL2002	*map*	Increased	map protein	

SACOL0095	*spa*	Decreased	immunoglobulin G binding protein A precursor-	Adhesion, virulence & diseases
SACOL2544	*sdaAA*	Decreased	Glycine, serine and threonine metabolism	Metabolism
SACOL2544	*ribD*	Decreased	Riboflavin metabolism	
SACOL2694	*geh*	Increased	Lipid metabolism (lipase)	
SACOL1329	*femC*	Decreased	Energy metabolism	
SACOL1818	*ribBA*	Decreased	Riboflavin biosynthesis	
SACOL1210	*pyrA*	Decreased	bifunctional pyrimidine regulatory protein PyrR uracil phosphoribosyltransferase	
SACOL1328	*glnR*	Decreased	Glutamine synthetase repressor	
SACOL0276	*yuKA*	Decreased	diarrheal toxin	

[A]FOF exposure = 0.5X MIC

[B]Locus tag for probe sequence present in COL genome, GenBank accession: CP000046.1

Table S2B. Summary of DAP-S 1483 gene functional classes altered ≥ 2-fold in DAP-S 1483 exposed by DAP[A] alone.

Locus Tag[B]	Gene Name	Expression Level	Pathway Descriptions	Related pathways
SACOL1328	*glnR*	Decreased	Glutamine synthetase repressor	
SACOL2628	*betB*	Decreased	Amino acid (betaine aldehyde dehydrogenase)	
SACOL2627	*betA*	Decreased	Amino acid (choline dehydrogenase)	
SACOL1215	*carB*	Decreased	carbamoyl-phosphate synthase (lipid metabolism)	
SACOL1214	*carA*	Decreased	carbamoyl-phosphate synthase (lipid metabolism)	
SACOL2703	*hisG*	Decreased	Histidine metabolism	
SACOL2700	*hisB*	Decreased	Histidine metabolism	
SACOL2129	*deoC2*	Decreased	Pentose phosphate pathway	
SACOL2656	*arcB2*	Decreased	Arginine biosynthesis	
SACOL0222	*ldh1*	Decreased	lactate dehydrogenase Glycolysis / Gluconeogenesis Pyruvate	
SACOL2482	*fabG2*	Decreased	3-oxoacyl-(acyl carrier protein) reductase Nucleic acid metabolism	
SACOL0154	*aldA1*	Decreased	Glycolysis / Gluconeogenesis	
SACOL1329	*femC*	Decreased	glutamine synthetase ; Glyoxylate and dicarboxylate metabolism, Alanine, aspartate and glutamate metabolism	
SACOL1742	*glta*	Decreased	Citrate synthase TCA cycle	Metabolism
SACOL1478	*ald1*	Decreased	alanine dehydrogenase	
SACOL2265	*mobB*	Decreased	molybdopterin-guanine dinucleotide biosynthesis adapter protein	
SACOL2029	*sacA*	Decreased	Galactose metabolism	
	ald2	Decreased	alanine dehydrogenase	
SACOL1782	*fhs*	Decreased	formate--tetrahydrofolate ligase; Carbon fixation pathways in prokaryotes	
SACOL1431	*dapB*	Decreased	4-hydroxy-tetrahydrodipicolinate reductase; Lysine biosynthesis	
SACOL1595	*gcvT*	Decreased	aminomethyltransferase; Glycine, serine and threonine metabolism ;Glyoxylate and dicarboxylate metabolism	
SACOL1385	*acnA*	Decreased	aconitate hydratase; TCA cycle	
SACOL1604	*glk*	Decreased	Glycolysis / Gluconeogenesis	
SACOL1741	*icd*	Decreased	Citrate cycle (TCA cycle)	
SACOL1506	*aroC*	Decreased	chorismate synthase; Phenylalanine, tyrosine and tryptophan biosynthesis	
SACOL0357	*dut*	Decreased	Pyrimidine metabolism; Drug metabolism	
SACOL0877	*gcvH*	Increased	Glycine, serine and threonine metabolism	

SACOL0148	*cap5M*	Decreased	capsular polysaccharide biosynthesis galactosyltransferase (Cell wall & capsule)	Cell wall and capsule synthesis
SACOL2632	*cudT*	Decreased	betaine/carnitine transporter	Transporter
SACOL2272	*modA*	Decreased	molybdate transport system substrate-binding protein	
SACOL2399	*nirR*	Decreased	transcriptional regulator	
SACOL2137	*czrA*	Decreased	transcriptional regulator; zinc-responsive transcriptional repressor	
SACOL1191	*mraZ*	Increased	transcriptional regulator; genetic information processing	
SACOL1587	*efp*	Increased	Elongation factor	Translation
SACOL2267	*moaC*	Increased	Translation factor	

[A]DAP exposure = 0.5X MIC

[B]Locus tag for probe sequence present in COL genome, GenBank accession: CP000046.1

Table S2C. Summary of gene functional classes altered ≥ 2-fold in DAP-S 1483 exposed by DAP-FOF vs. DAP[A] alone

Locus Tag[B]	Gene Name	Expression Level	Pathway Descriptions	Related Pathways
SACOL0137	*cap5B*	Decreased	capsular polysaccharide biosynthesis protein (Cell wall & capsule)	Cell wall and capsule synthesis
SACOL0138	*cap5C*	Decreased	capsular polysaccharide biosynthesis protein (Cell wall & capsule)	
SACOL0139	*cap5D*	Decreased	capsular polysaccharide biosynthesis protein (Cell wall & capsule)	
SACOL0140	*cap5E*	Decreased	capsular polysaccharide biosynthesis protein (Cell wall & capsule)	
SACOL0141	*cap5F*	Decreased	capsular polysaccharide biosynthesis protein (Cell wall & capsule)	
SACOL0142	*cap5G*	Decreased	capsular polysaccharide biosynthesis protein (Cell wall & capsule)	
SACOL0143	*cap5H*	Decreased	capsular polysaccharide biosynthesis protein (Cell wall & capsule)	
SACOL0144	*cap5I*	Decreased	capsular polysaccharide biosynthesis protein (Cell wall & capsule)	
SACOL0145	*cap5J*	Decreased	capsular polysaccharide biosynthesis protein (Cell wall & capsule)	
SACOL0146	*cap5K*	Decreased	capsular polysaccharide biosynthesis protein (Cell wall & capsule)	
SACOL0147	*cap5L*	Decreased	capsular polysaccharide biosynthesis protein	
SACOL0148	*cap5M*	Decreased	capsular polysaccharide biosynthesis galactosyltransferase (Cell wall & capsule)	
SACOL0149	*cap5N*	Decreased	capsular polysaccharide biosynthesis protein	
SACOL0150	*cap5O*	Decreased	capsular polysaccharide biosynthesis protein (Cell wall & membrane biogenesis)	
SACOL0151	*cap5P*	Decreased	UDP-N-acetylglucosamine 2-epimerase – (Cell wall & membrane biogenesis)	
SACOL0610	*sdrE*	Decreased	Serine-aspartate repeat-containing protein E	Adhesion, pathogenesis, bacterial clearance and infection
SACOL2505	sas*G*	Increased	Cell wall surface anchor family protein	
SACOL2656	*arcB2*	Increased	ornithine carbamoyl transferase	Metabolism
SACOL2324	*hutU*	Increased	urocanate hydratase	
SACOL2507	sarU	Increased	Staphylococcal accessory regulator; activator of *agr* expression	Regulators
SACOL2326	*fosB*	Increased	fosfomycin resistance protein	Cell Envelope
SACOL0095	*spa*	Increased	immunoglobulin G binding protein A precursor-	Virulence/Infection
SACOL0247	*lrgA*	Decreased	murein hydrolase regulator LrgA -	Transporters
SACOL0248	*lrgB*	Decreased	antiholin-like protein LrgB-	

[A]DAP and FOF exposures = 0.5X MIC for each antibiotic

[B]Locus tag for probe sequence present in COL genome, GenBank accession: CP000046.1

Table S2D: Summary of gene functional classes altered ≥ 2-fold in DAP-R 185 exposed by FOF[A]

Locus Tag[B]	Gene Name	Expression Level	Pathway Descriptions	Related Pathway
SACOL2627	*betA*	Decreased	Amino acid (choline dehydrogenase)	Metabolism
SACOL2628	*betB*	Decreased	Amino acid (betaine aldehyde dehydrogenase)	
SACOL0263	*lytM*	Decreased	Peptidoglycan biosynthesis and degradation proteins	
SACOL0494	*nuoF*	increased	Energy metabolism	
SACOL1056	*sspB1*	Decreased	staphopain B; peptidase and inhibitors	
SACOL1062	*atl*	Decreased	Glycan biosynthesis	
SACOL2323	*hutL*	increased	Histidine metabolism	
SACOL0671	*dut*	Decreased	Nucleotide metabolism; drug metabolism	
SACOL2324	*hutU*	Increased	Histidine metabolism	
SACOL0247	*lrgA*	Decreased	murein hydrolase regulator	Transporter
SACOL0248	*lrgB*	Decreased	murein hydrolase regulator	
SACOL2632	*cudT*	Decreased	BCCT family osmoprotectant transporter	
SACOL2067	*kdpB*	Decreased	potassium-transporting ATPase ATP-binding subunit	
SACOL0137	*cap5B*	Decreased	capsular polysaccharide biosynthesis protein (Cell wall & capsule)	Cell wall and capsule synthesis
SACOL0138	*cap5C*	Decreased	capsular polysaccharide biosynthesis protein (Cell wall & capsule)	
SACOL0139	*cap5D*	Decreased	capsular polysaccharide biosynthesis protein (Cell wall & capsule)	
SACOL0140	*cap5E*	Decreased	capsular polysaccharide biosynthesis protein (Cell wall & capsule)	
SACOL0141	*cap5F*	Decreased	capsular polysaccharide biosynthesis protein (Cell wall & capsule)	
SACOL0142	*cap5G*	Decreased	capsular polysaccharide biosynthesis protein (Cell wall & capsule)	
SACOL0143	*cap5H*	Decreased	capsular polysaccharide biosynthesis protein (Cell wall & capsule)	
SACOL0144	*cap5I*	Decreased	capsular polysaccharide biosynthesis protein (Cell wall & capsule)	
SACOL0145	*cap5J*	Decreased	capsular polysaccharide biosynthesis protein (Cell wall & capsule)	
SACOL0146	*cap5K*	Decreased	capsular polysaccharide biosynthesis protein (Cell wall & capsule)	
SACOL0147	*cap5L*	Decreased	capsular polysaccharide biosynthesis protein	
SACOL0148	*cap5M*	Decreased	capsular polysaccharide biosynthesis galactosyltransferase (Cell wall & capsule)	
SACOL0149	*cap5N*	Decreased	capsular polysaccharide biosynthesis protein	

SACOL0150	*cap5O*	Decreased	capsular polysaccharide biosynthesis protein (Cell wall & membrane biogenesis)	
SACOL0151	*cap5P*	Decreased	UDP-N-acetylglucosamine 2-epimerase – (Cell wall & membrane biogenesis)	
SACOL2676	*sasA*	Decreased	Cell wall surface anchor protein	
SACOL2326	*fosB*	Increased	fosfomycin resistance protein	
SACOL0856	*clfA*	Decreased	clumping factor A; *S. aureus* infection	VirulenceInfection
SACOL0610	*sdrE*	Decreased	clumping factor A; *S. aureus* infection	
SACOL0276	*yukA*	Decreased	*S. aureus* infection	Translation
SACOL0979	*clpB*	Decreased	genetic information processing	
SACCOL1254	*rpsP*	Increased	genetic information processing; Chaperones and folding catalysts	

[A]FOF exposure = 0.5X MIC

[B]Locus tag for probe sequence present in COL genome, GenBank accession: CP000046.1

Table S2E. Summary of gene functional classes altered ≥ 2-fold in DAP-R 185 exposed by DAP[A]

Locus Tag[B]	Gene Name	Expression level	Pathway Descriptions	Related Pathway
SACOL0204	*pflB*	Decreased	formate acetyltransferase; Pyruvate, propanoate, Butanoate metabolism	
SACOL0205	*pflA*	Decreased	pyruvate formate-lyase-activating enzyme; Pyruvate, metabolism	
SACOL2398	*nirB*	Decreased	Nitrite reductase (NADH) large subunit ; Nitrogen metabolism	
SACOL2394	*narH*	Decreased	Nitrogen metabolism	
SACOL2628	*betB*	Decreased	betaine-aldehyde dehydrogenase	
SACOL1782	*fhs*	Decreased	formate--tetrahydrofolate ligas; Carbon fixation pathways in prokaryotes	
SACOL2281	*ureB*	Decreased	Arginine biosynthesis, Purine metabolism	
SACOL2083	*thiE*	Decreased	thiamine-phosphate pyrophosphorylase; Thiamine metabolism	
SACOL2577	*crtM*	Decreased	Carotenoid biosynthesis	
SACOL2084	*thiM*	Decreased	Thiamine metabolism	
SACOL2627	*betA*	Decreased	choline dehydrogenase	
SACOL2085	*thiD2*	Decreased	Thiamine metabolism	
SACOL0222	*ldh1*	Decreased	Glycolysis / Gluconeogenesis; Pyruvate metabolism	
SACOL0154	*aldA1*	Decreased	Glycolysis / Gluconeogenesis	
SACOL1408	*trpB*	Decreased	Biosynthesis of amino acids	Metabolism
SACOL2129	*deoC2*	Decreased	deoxyribose-phosphate aldolase; Carbohydrate/pentose phosphate metabolism	
SACOL2048	*leuC*	Decreased	Biosynthesis of amino acids; Glucosinolate biosynthesis	
SACOL0357	*dut*	Decreased	prophage L54a, deoxyuridine 5'-triphosphate nucleotidohydrolase	
SACOL0263	*lytM*	Decreased	peptidoglycan hydrolase; Peptidoglycan biosynthesis and degradation protein	
SACOL0517	*treC*	Decreased	trehalose-6-phosphate hydrolase; Carbohydrate metabolism	
SACOL0545	*rplY*	Decreased	Stress response	
SACOL2415	*gpm*	Decreased	phosphoglycerate mutase; Glycolysis / Gluconeogenesis	
SACOL0693	*tagA*	Decreased	N-acetylglucosaminyldiphosphoundecaprenol N-acetyl-beta-D-mannosaminyltransferase; Teichoic acid biosynthesis	
SACOL1374	*lexA*	Decreased	repressor LexA; DNA repair and recombination proteins	
SACOL1210	*pyrR*	Decreased	Pyrimidine metabolism	

SACOL_Sa23SD	*rrlD*	Decreased	Genetic information processing	Translation
SACOL_Sa23SB	*rrlB*	Decreased	Genetic Information Processing	
SACOL_Sa23SE	*rrlE*	Decreased	Genetic Information Processing	
SACOL_Sa16SF	*rrsF*	Decreased	Genetic information processing	
SACOL_Sa16SC	*rrsC*	Decreased	Genetic information processing	
SACOL_Sa16SA	*rrsA*	Decreased	Genetic information processing	
SACOL_Sa16SB	*rrsB*	Decreased	Genetic information processing	
SACOL_Sa16SD	*rrsD*	Decreased	Genetic information processing	
SACOL1637	*dnaK*	Decreased	molecular chaperone	
SACOL1254	*rpsP*	Decreased	Genetic Information Processing	
SACOL1257	*rplS*	Decreased	Genetic Information Processing	
SACOL1370	*rpsN1*	Decreased	Genetic Information Processing	
SACOL1642	*rpsT*	Decreased	Genetic Information Processing	
SACOL0586	*rplL*	Decreased	Genetic Information Processing	
SACOL0585	*rplJ*	Decreased	Genetic Information Processing	
SACOL0691	*sirR*	Decreased	transcriptional regulator, Mn-dependent transcriptional regulator	Transcription
SACOL2287	*sarR*	Decreased	accessory regulator	
SACOL2384	*sarZ*	Decreased	accessory regulator	
SACOL2399	*nirR*	Decreased	transcriptional regulator	
SACOL2421	*hlgC*	Decreased	hemolysin toxin family protein	Virulence/Infection
SACOL0856	*clfA*	Decreased	clumping factor A	
SACOL2016	*groEL*	Decreased	RNA degradation	
SACOL0570	*clpC*	Decreased	Virulence regulation	
SACOL2067	*kdpB*	Decreased	potassium-transporting ATPase ATP-binding subunit	Transporter
SACOL2068	*kdpA*	Decreased	Potassium-transporting ATPase potassium-binding subunit	
SACOL2066	*kdpC*	Decreased	potassium-transporting ATPase KdpC subunit	
SACOL2070	*kdpD*	Decreased	sensor histidine kinase	
SACOL0099	*sirA*	Decreased	iron compound ABC transporter iron compound-binding protein	
SACOL2422	*hlgB*	Decreased	hemolysin toxin family protein	Toxin
SACOL1173	*hlY*	Decreased	Hemolysin II	
SACOL0137	*cap5B*	Decreased	capsular polysaccharide biosynthesis protein (Cell wall & capsule)	
SACOL0138	*cap5C*	Decreased	capsular polysaccharide biosynthesis protein (Cell wall & capsule)	
SACOL0139	*cap5D*	Decreased	capsular polysaccharide biosynthesis protein (Cell wall & capsule)	
SACOL0140	*cap5E*	Decreased	capsular polysaccharide biosynthesis protein (Cell wall & capsule)	
SACOL0141	*cap5F*	Decreased	capsular polysaccharide biosynthesis protein (Cell wall & capsule)	

SACOL0142	*cap5G*	Decreased	capsular polysaccharide biosynthesis protein (Cell wall & capsule)	Cell wall and capsule synthesis
SACOL0143	*cap5H*	Decreased	capsular polysaccharide biosynthesis protein (Cell wall & capsule)	
SACOL0144	*cap5I*	Decreased	capsular polysaccharide biosynthesis protein (Cell wall & capsule)	
SACOL0145	*cap5J*	Decreased	capsular polysaccharide biosynthesis protein (Cell wall & capsule)	
SACOL0146	*cap5K*	Decreased	capsular polysaccharide biosynthesis protein (Cell wall & capsule)	
SACOL0147	*cap5L*	Decreased	capsular polysaccharide biosynthesis protein (Cell wall & capsule)	
SACOL0148	*cap5M*	Decreased	capsular polysaccharide biosynthesis galactosyltransferase (Cell wall & capsule)	
SACOL0149	*cap5N*	Decreased	capsular polysaccharide biosynthesis protein (Cell wall & capsule)	
SACOL0150	*cap5O*	Decreased	capsular polysaccharidebiosynthesis protein (Cell wall & membrane biogenesis)	

[A]DAP exposure = 0.5X MIC

[B]Locus tag for probe sequence present in COL genome, GenBank accession: CP000046.1

Table S2F. Summary of gene functional classes altered ≥ 2-fold in DAP-R 185 exposed by DAP + FOF vs DAP[A] alone

Locus Tag[B]	Gene Name	Expression Level	Pathway Descriptions	Related Pathway
SACOL_S23SD	*rrlD*	Increased	Ribosomal RNA, 16S	Translation
SACOL_Sa16SB	*rrsB*	Increased	Ribosomal RNA, 16S	
SACOL_Sa16SA	*rrsA*	Increased	Ribosomal RNA, 16S	
SACOL_Sa16SC	*rrsC*	Increased	Ribosomal RNA, 16S	
SACOL_Sa16SF	*rrsF*	Increased	Ribosomal RNA, 16S	
SACOL_Sa16SD	*rrsD*	Increased	Ribosomal RNA, 16S	
SACOL_Sa23SE	*rrlE*	Increased	Ribosomal RNA, 23S	
SACOL_Sa23SB	*rrlB*	Increased	Ribosomal RNA, 23S	
SACOL2326	fosB	Increased	fosfomycin resistance protein	Cell Envelope
SACOL0247	*lrgA*	Decreased	murein hydrolase regulator LrgA -	Transporter
SACOL0248	*lrgB*	Decreased	antiholin-like protein LrgB	
SACOL2628	*betB*	Decreased	Amino acid (betaine aldehyde dehydrogenase)	Metabolism
SACOL2699	*hisH*	Decreased	Hydrolase, lyase Imidazole glycerol phosphate synthase subunit HisH	
SACOL2394	*narH*	Decreased	Microbial metabolism in diverse environments	
SACOL2395	*narG*	Decreased	Microbial metabolism in diverse environments	
SACOL1816	*putA*	Decreased	proline dehydrogenase	
SACOL1406	*trpC*	Decreased	indole-3-glycerol phosphate synthase	
SACOL0610	*sdrE*	Decreased	Serine-aspartate repeat-containing protein E	Adhesion, pathogenesis, bacterial clearance, and infection

[A]DAP and FOF exposures = 0.5x MIC for each antibiotic

[B]Locus tag for probe sequence present in COL genome, GenBank accession: CP000046.1

Table S3. Primers used in this study for qRT-PCR gene expression analyses.

Gene Name	Primer Sequences
gyrB	Fwd 5’ CGCAGGCGATTTTACCATTA<br>Rev 5' GCTTTCGCTAGATCAAAGTCG
lrgB	Fwd 5’ TCG GAG GTA TTG GTA TCG<br>Rev 5’ CTG CTT GAG GTA ACA TTG A
